# Assessment of Dietary and Lifestyle Responses After COVID-19 Vaccine Availability in Selected Arab Countries

**DOI:** 10.3389/fnut.2022.849314

**Published:** 2022-04-14

**Authors:** Leila Cheikh Ismail, Tareq M. Osaili, Maysm N. Mohamad, Amina Al Marzouqi, Carla Habib-Mourad, Dima O. Abu Jamous, Habiba I. Ali, Haleama Al Sabbah, Hayder Hasan, Hussein Hassan, Lily Stojanovska, Mona Hashim, Muna AlHaway, Radwan Qasrawi, Reyad R. Shaker Obaid, Rameez Al Daour, Sheima T. Saleh, Ayesha S. Al Dhaheri

**Affiliations:** ^1^Department of Clinical Nutrition and Dietetics, College of Health Sciences, University of Sharjah, Sharjah, United Arab Emirates; ^2^Nuffield Department of Women’s & Reproductive Health, University of Oxford, Oxford, United Kingdom; ^3^Department of Nutrition and Food Technology, Faculty of Agriculture, Jordan University of Science and Technology, Irbid, Jordan; ^4^Department of Nutrition and Health, College of Medicine and Health Sciences, United Arab Emirates University, Al Ain, United Arab Emirates; ^5^Department of Health Services Administration, College of Health Sciences, University of Sharjah, Sharjah, United Arab Emirates; ^6^Department of Nutrition and Food Sciences, Faculty of Agricultural and Food Sciences, American University of Beirut, Beirut, Lebanon; ^7^Research Institute of Medical & Health Sciences, University of Sharjah, Sharjah, United Arab Emirates; ^8^College of Natural and Health Sciences, Zayed University, Dubai, United Arab Emirates; ^9^Department of Natural Sciences, School of Arts and Sciences, Lebanese American University, Beirut, Lebanon; ^10^Institute for Health and Sport, Victoria University, Melbourne, VIC, Australia; ^11^Blood Transfusion and Research Center, Emirates Health Services, Dubai, United Arab Emirates; ^12^Department of Computer Science, Al-Quds University, Jerusalem, Palestine; ^13^Department of Computer Engineering, Istinye University, Istanbul, Turkey

**Keywords:** Arab countries, COVID-19 pandemic, COVID-19 vaccination, dietary habits, lifestyle behaviors

## Abstract

**Background:**

The COVID-19 pandemic has been consistently associated with unhealthy lifestyle behaviors and dietary practices. This study aimed to assess the dietary and lifestyle behaviors of adults after COVID-19 vaccine availability and their attitude toward the vaccine in selected Arab countries.

**Methods:**

A cross-sectional survey-based study was conducted between October 2021 and December 2021 using Google Forms (*n* = 2259). A multi-component questionnaire was used to collect socio-demographic characteristics, attitudes toward the COVID-19 vaccine, and behavioral, dietary, and lifestyle responses after easing the restriction. Participants were given a score based on the sum of positive dietary and lifestyle changes. The generalized linear models were used to identify the association between positive dietary and lifestyle changes score and sociodemographic characteristics.

**Results:**

Weight gain during the pandemic was reported by 39.5% of the participants, 36.1% reported ever getting infected with the COVID-19 virus, and 85% received at least one dose of the vaccine. The key adverse reactions of the COVID-19 vaccine were fatigue, headache, and joint pain, and the main reason for vaccination was protection against infection. Most participants were concerned about the vaccine side effects (45.8%) and inadequate testing (50.7%). After easing of restriction, 54.3% of the participants reduced the frequency of disinfecting objects, and 58.3% joined social events. Most dietary and lifestyle behaviors remained unchanged after vaccine availability but there was an increase in the time spent behind the screen for work (50.1%) and entertainment (42.9%). The results of the multivariate regression analyses revealed that older participants (*p* = 0.001), those with higher education (*p* = 0.010), and those working from home (*p* = 0.040) were more likely to have higher positive dietary and lifestyle changes scores.

**Conclusion:**

Although most participants were concerned about vaccine safety, low vaccine hesitancy rates were observed among the study sample. The availability of the COVID-19 vaccines resulted in loosening some of the safety social measures among Arab adults but the negative impact of the pandemic on dietary and lifestyle behaviors remained unaltered.

## Introduction

The novel coronavirus 2019 (COVID-19) pandemic caused by the SARS-CoV-2 virus is far from resolved as the virus is constantly changing through mutations, and new variants have been detected across the globe (1). More transmissible variants of the virus, those that may increase disease severity, or may decrease vaccine effectiveness are referred to as variants of concern (VOCs) (2). Since December 2020, five VOCs have been detected including Alpha, Beta, Gamma, Delta, and Omicron (1). Thus, the number of new cases is still surging around the globe posing an increased risk to global public health. As the effectiveness of the vaccines against VOC is still under investigation (3), public health authorities, such as the World Health Organization (WHO) encourage countries to continue implementing the precautious existing public health and social measures.

In the early stages of the COVID-19 pandemic, countries were forced to act promptly due to the absence of a cure or a vaccine, and apply restrictions and safety measures to contain the spread of the virus by focusing on changing public behavior (4). Preventive non-pharmaceutical interventions (NPIs) varied from mandating face masks and social distancing to tougher measures including complete lockdowns, isolation of the infected population, teleworking, and virtual education. Several countries in the Middle East enforced complete or partial lockdowns by fines and penalties such as the United Arab Emirates and Jordan (5). Although these strict measures were effective in preventing and delaying the spread of the virus, they entail enormous socio-economic costs and have negatively impacted the quality of life (6). Our previous research in the Middle East and North Africa (MENA) region revealed that lockdowns were associated with a variety of negative lifestyle and dietary habits, physical inactivity, high screen time, sleep disturbances, and anxious psychological feelings among adults (7–10).

Vaccination against COVID-19 is one of the most effective ways to contain the infection. By December 2020, the WHO approved the use of Pfizer/BioNTech for emergency (11) and other vaccines including AstraZeneca/Oxford, Johnson and Johnson, Moderna, Sinopharm, Sinovac, and COVAXIN were deemed safe and efficient by the WHO during 2021 (12). Within 1 year, around 8.8 billion vaccine doses were administered globally and 48.3% of the world population has been fully vaccinated against COVID-19 (13). In the MENA region, up to 91% of the population in the United Arab Emirates, 38% in Jordan, 29% in Palestinian territories, and 28% in Lebanon were fully vaccinated against COVID-19 by the end of 2021 (14). With the increasing vaccination rates, countries were able to gradually lift some of the NPIs including lockdowns, travel bans, and capacity restrictions on gatherings (15). It was believed that adherence to preventive measures such as hygiene behaviors might be reduced after easing the restrictions and the availability of the vaccine, while adherence to social distancing and masking may remain high (16). However, behavioral and lifestyle changes after the availability of the vaccine have not been investigated in Arab countries yet.

Apart from the Arab Gulf countries, vaccine rates in the MENA region remain low (13). A recent systematic review found low acceptance rates of the COVID-19 vaccines in the Middle East (17): with Lebanon (21%) (18), Jordan (37.4%) (19), United Arab Emirates (60%) (20), and Palestine territories (63%) (21). Thus, vaccine hesitancy is posing crucial challenges in controlling the COVID-19 pandemic. Several studies investigated vaccine acceptability among the public and found that the most common factors for willingness to get the vaccine were self-protection and stopping the spread of the virus (22). In the United Arab Emirates and Jordan, the main motivators for vaccine acceptability included the safety and efficacy of the vaccine, followed by a low risk of side effects, and higher overall protection (23, 24). A study among university students in Lebanon revealed that a lower level of knowledge about the COVID-19 disease was associated with higher vaccine hesitancy (25). With numerous conspiracies about the vaccine on social media platforms, it is critical to investigate attitudes toward the vaccine and the reasons behind the willingness to get vaccinated.

Since the onset of the COVID-19 pandemic, numerous studies have evaluated its impact on dietary and lifestyle behaviors globally (26, 27) and in Arab countries (9, 28–31). In Canada, a quarter of participants reported an increase in the consumption of junk food during the early stage of the pandemic (32). A recent review has indicated an increase in the consumption of unhealthy foods such as fried food, sugar-added drinks, and processed meat during home confinement while consumption of fruit and vegetable was reduced (33). In the United Arab Emirates, results suggested an increased food intake, weight gain, higher smoking rate, sedentary time, and sleep disturbances (28, 29). Similarly, in Lebanon, unhealthy eating habits were prevalent among adults including low intake of water, fruits, and vegetables (9). Moreover, adults in Jordan and Palestinian territories reported increased consumption of meals and snacks during the COVID-19 pandemic (30, 31).

However, most studies evaluating the impact of the pandemic on eating habits were conducted during the early stages of the pandemic and lockdowns. A longitudinal study in the United Kingdom suggested fluctuations in dietary habits during the first year of the pandemic with a persistent decrease in the consumption of fruits and vegetables (34). Limited data is available on the dietary changes and lifestyle behaviors that might have been retained after the availability of the vaccine and relaxation of NPIs. Moreover, it would be important to investigate whether people have gained new habits during the pandemic that are sustainable in the future. Therefore, this study aims to assess the dietary and lifestyle responses after COVID-19 vaccine availability and to ascertain attitudes toward the vaccine in selected Arab countries.

## Materials and Methods

### Study Design

This cross-sectional survey-based study was conducted in selected Arab countries between October 2021 and December 2021. A convenience sample approach was adopted where adults from the United Arab Emirates, Lebanon, Palestine territories, and Jordan were invited to participate. A web link to the online survey was disseminated *via* e-mail invitations and social media platforms, e.g., LinkedIn™, Facebook™, and WhatsApp™. An information sheet explaining the objective and study protocol was offered as the first page of the survey, and participants were required to consent and verify their age and country of residence before proceeding to the questionnaire. To reduce potential sampling bias, participants were encouraged to pass on the questionnaire to a maximum of three individuals from different households.

This study was performed in compliance with the ethical code for web-based research (35) and in line with principles presented in the Declaration of Helsinki. The study protocol received Ethical Approval from the University of Sharjah Research Ethics Committee (Ref: REC-21-10-27-1) and the Institutional Review Board of the Jordan University of Science and Technology (Ref.: 33/142/2021).

### Participants

The criterion for participation in the study was living in the United Arab Emirates, Lebanon, Palestine territories, or Jordan and aged 18 years or older. There were no restrictions on age, gender, education, vaccination, or type of COVID-19 vaccine.

A total of 2,259 participants completed the questionnaire from four Arab countries: Jordan (22.9%), Lebanon (25.9%), Palestine territories (27.7%), and United Arab Emirates (23.5%). The data were collected and analyzed anonymously to maintain confidentiality, and electronic informed consent was obtained from all participants. Participants were not rewarded for completing the online survey and were free to withdraw at any point. Only completed questionnaires were saved into the system and were included in the analysis of the study.

### Questionnaire

A multicomponent, self-administered online questionnaire was developed using Google Forms in English and Arabic. The first draft of the questionnaire was developed by researchers at the University of Sharjah based on relevant literature and our previous study in the MENA region (7, 36). The questions were then reviewed and validated by a panel of experts for content relevance.

The questionnaire was originally developed in the English language and forward translated into Arabic by a bilingual translation expert. It was then backward translated into English by a different bilingual translation expert. The questionnaire required an estimated time of 10–15 min to complete. It was pilot tested with 30 people in the United Arab Emirates, Lebanon, and Jordan, to assess the clarity of the questions, and no significant modifications were required. The pilot-testing data was not included in the results of the study. The internal consistency of the questionnaire was evaluated by calculating the Cronbach’s α coefficient. The questionnaire in this study was shown to be a reliable instrument as indicated by a Cronbach’s alpha of 0.81, which suggests a good internal consistency (37).

The questionnaire was divided into four sections: (Section 1) Socio-demographic characteristics (11 items): age, sex, marital status, education level, employment status, work or study setting, weight change during the pandemic, medical history, previous infection of COVID-19, COVID-19 vaccine status, and country of residence. Those who got vaccinated were further asked about adverse reactions after getting the vaccine and reasons to take the vaccine; (Section 2) Attitudes toward the COVID-19 vaccine (9 items): the seriousness of the COVID-19 pandemic, understandability, feeling of control, vulnerable groups and their risk of infection, COVID-19 vaccine safety and efficacy questions; (Section 3) Behavioral responses after easing the restriction (8 items): avoidance of places and activities that pose a risk of infection and compliance with recommended activities to decrease the risk of infection; (Section 4) Dietary and lifestyle changes after easing the restrictions (18 items): meal type, food intake, intake of immune-boosting foods or supplements, number of meals per day, food choices, number of meals consumed with family or friends, breakfast consumption, skipping meals, snacking, water intake, physical activity, screen time for work/leisure, sleep quality and energy level. The full version of the questionnaire can be found in the Supplementary Material.

### Data Analysis

Categorical variables are presented as frequencies and percentages and continuous variables were presented as means (M) and standard deviations (SD). The Chi-Square test (χ^2^) test was used to examine attitude differences by country. Each participant was given a score based on the sum of positive dietary and lifestyle changes. Favorable dietary and lifestyle changes included: increased consumption of fruits and vegetables (vs. same or decreased), decreased consumption of fast foods (vs. same or increased), decreased consumption of fried foods (vs. same or increased), increased number of meals consumed with family or friends (vs. same or decreased), consume breakfast daily, do not skip meals, drinking ≥2 l of water per day, increased physical activity (vs. same or decreased), decreased screen time for entertainment (vs. same or increased), improved sleep quality (vs. same or worsened), and improved level of energy (vs. same or worsened). Positive dietary and lifestyle changes score was calculated whereby participants will receive 0–11 points based on the number of favorable dietary and lifestyle changes they reported. Each variable was counted as 1 point toward the overall score and the sum was calculated for each participant. A higher score indicated a high number of positive dietary and lifestyle changes. The generalized linear model analyses were carried out to investigate the association between the positive dietary and lifestyle changes score and sociodemographic characteristics. The variables entered in the final multivariate regression model were selected with the use of a univariate general linear model, with the use of a cut-off value of *p* < 0.05 to be included. Statistical analyses were carried out using the Statistical Package for the Social Sciences (SPSS Inc., IBM, Chicago, IL, United States) version 26.0. A *p*-value of less than 0.05 was considered statistically significant. As the data was collected through a web link and all questions were required, no missing values were recorded.

## Results

### Demographic Characteristics

Key demographic variables of the study population are presented in Table 1. Participants’ ages ranged from 18 to 83 years (*M* = 31.1, SD = 12.6), with 23.7% males. Most surveyed individuals were single (52.6%), completed a university degree (56.9%), worked full-time (33.7%), and were not working/studying from home (59.1%).

**TABLE 1:** Demographic breakdown of surveyed participants (*n* = 2259).

Characteristics	
**Age (years), mean (SD)**	31.1 (12.6)
**Sex, *n* (%)**	
Male	535 (23.7)
Female	1,724 (76.3)
**Marital status, *n* (%)**	
Married	1,012 (44.8)
Single	1,189 (52.6)
Divorced	40 (1.8)
Widowed	18 (0.8)
**Education level, *n* (%)**	
Less than high school	64 (2.8)
High school	217 (9.6)
College/Diploma	317 (14.0)
Bachelor’s degree	1,285 (56.9)
Higher than bachelor’s degree	376 (16.6)
**Employment status, *n* (%)**	
Full-time	761 (33.7)
Part-time	123 (5.4)
Self-employed	153 (6.8)
Student	667 (29.5)
Unemployed	495 (21.9)
Retired	60 (2.7)
**Working/studying from home, *n* (%)**	
Yes	711 (31.5)
No	1,334 (59.1)
Not applicable	214 (9.5)
**Weight change during the pandemic, *n* (%)**	
Lost weight	451 (20)
Gained weight	893 (39.5)
Maintained weight	915 (40.5)
**Have chronic disease, *n* (%)**	
Yes	244 (10.8)
No	2,015 (89.2
**Have ever been infected by the COVID-19 virus, *n* (%)**	
Yes	815 (36.1)
No	1,444 (63.9)
**Received the COVID-19 vaccine, *n* (%)**	
Yes (≥2-doses)	1,753 (77.6)
Yes (1-dose)	178 (7.9)
No (but planning to take it)	149 (6.6)
No (I do not want to take it)	179 (7.9)
**Country of residence, *n* (%)**	
United Arab Emirates	530 (23.5)
Jordan	517 (22.9)
Lebanon	586 (25.9)
Palestine territories	626 (27.7)

*Values represent frequencies and percentages [n (%)] or mean and standard deviation [mean (SD)].*

Over one-third of the respondents reported weight gain during the pandemic (39.5%), while 20% lost weight, and 40.5% maintained weight. Only 11% of the respondents had chronic conditions, and 36.1% reported ever getting infected with the COVID-19 virus. Over 85% received at least one dose of the vaccine, 77.6% received two or more doses of the vaccine, and only 7.9% had no desire to get vaccinated.

**FIGURE 1:**
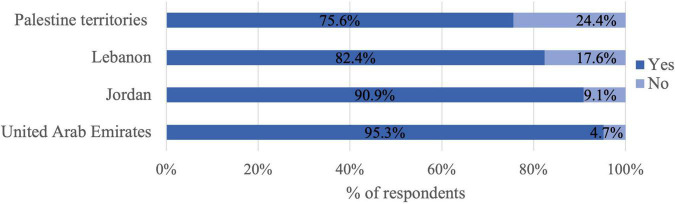
Percentage of participants who have received at least one dose by country (*n* = 2295).

As shown in Figure 1, the highest percentage of participants who received at least one dose of the COVID-19 vaccines was reported in the United Arab Emirates (95.3%), followed by Jordan (90.9%), Lebanon (82.4%), and Palestine territories (75.6%).

**FIGURE 2:**
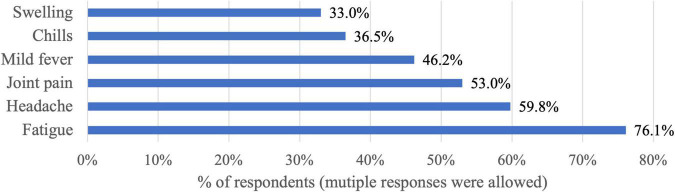
The main stated adverse reactions after receiving the COVID-19 vaccine (*n* = 1931).

Of the participants who received at least one dose of the vaccine, 63.5% reported experiencing adverse reactions. The main adverse reaction was fatigue (76.1%), followed by headache (59.8%), and joint pain (53%), and the least stated side effect was swelling of the arm (33%) (Figure 2).

**FIGURE 3:**
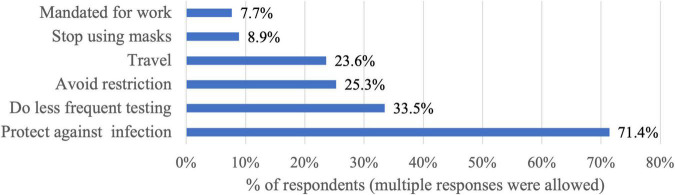
The motivators to get COVID-19 vaccination (*n* = 1931).

The participants were asked about the reasons behind getting the vaccine against COVID-19 and varied responses were obtained (Figure 3). The top reported reasons for vaccination were to protect against infection (71.4%), to get tested less frequently (33.5%), and to avoid restrictions (25.3%). The least selected reason was that the vaccine is mandated for work purposes (7.7%).

### Attitude Toward the COVID-19 Vaccine

Most of the participants (44.8%) believe the current COVID-19 situation is serious and 84.5% of the participants were aware that the pandemic is far from being over (Table 2). Most of the participants reported that not only those who have underlying medical problems should be vaccinated (71.6%). However, the majority were concerned about the side effects of the COVID-19 vaccine (45.8%) and that the vaccine has not been tested adequately (50.7%).

**TABLE 2:** Attitude toward the COVID-19 vaccine (*n* = 2259).

Attitudes	*n* (%)
**I think the current COVID-19 situation is serious.**	
Agree	1,013 (44.8)
Neutral	915 (40.5)
Disagree	331 (14.7)
**I do not understand what is happening with the COVID-19 pandemic.**	
Agree	610 (27.0)
Neutral	831 (36.8)
Disagree	818 (36.2)
**I think that whether I get the coronavirus or not is out of my control**	
Agree	1,140 (50.5)
Neutral	680 (30.1)
Disagree	439 (19.4)
**In my opinion, people are still going to be catching the coronavirus**	
Agree	1,909 (84.5)
Neutral	147 (6.5)
Disagree	203 (9.0)
**Only people who have underlying medical problems should be vaccinated**	
Agree	269 (11.9)
Neutral	372 (16.5)
Disagree	1,618 (71.6)
**The COVID-19 vaccine will protect me from coronavirus infection.**	
Agree	776 (34.4)
Neutral	766 (33.9)
Disagree	717 (31.7)
**I am concerned about the side effects of the COVID-19 vaccine.**	
Agree	1,035 (45.8)
Neutral	688 (30.5)
Disagree	536 (23.7)
**I am concerned that the vaccine has not been tested adequately.**	
Agree	1,146 (50.7)
Neutral	688 (30.5)
Disagree	425 (18.8)
**The COVID-19 vaccine will stop the spread of coronavirus.**	
Agree	667 (29.5)
Neutral	844 (37.4)
Disagree	748 (33.1)

### Behavioral Responses After Easing the Restriction

Safety behaviors after vaccine availability and relaxation of restrictions are presented in Table 3. Fewer than one-third of the participants reported washing their hands less often (26.3%) and increased their use of public transportation (29.2%). More than half of the sample reduced the frequency of disinfecting objects (54.3%) and joined social events (58.3%). Participants from Palestine were more likely to reduce the use of sanitizers and increase the use of public transportation (χ^2^ = 45.74, *p* < 0.001). Whereas those living in Lebanon were more likely to be in crowded places and join social events (χ^2^ = 48.69, χ^2^ = 19.56, respectively, *p* < 0.001). Participants residing in the United Arab Emirates reported going shopping in stores more than before and sending their children to school or pre-school (χ^2^ = 32.53, χ^2^ = 19.85, respectively, *p* < 0.001).

**TABLE 3:** Behavioral responses after easing the restriction by country (*n* = 2259).

After easing the restriction, I have	Total (*n* = 2259)	United Arab Emirates (*n* = 530)	Jordan (*n* = 517)	Palestine (*n* = 626)	Lebanon (*n* = 586)	χ ^2^	*p*-value
			
	*n* (%)						
Washed my hands less often than usual with soap and water	595 (26.3)	155 (29.2)	122 (23.6)	167 (26.7)	151 (25.8)	4.45	0.217
Used alcoholic hand gel less than usual	1062 (47.0)	227 (42.8)	217 (42.0)	366 (58.5)	252 (43.0)	45.74	<0.001
Reduced the amount I clean or disinfect objects that I might touch	1226 (54.3)	275 (51.9)	285 (55.1)	351 (56.1)	315 (53.8)	2.25	0.523
Been in crowded places generally	994 (44.0)	214 (40.4)	170 (32.9)	311 (49.7)	299 (51.0)	48.69	<0.001
Increased the amount I use public transport	660 (29.2)	98 (18.5)	100 (19.3)	287 (45.8)	175 (29.9)	137.69	<0.001
Joined more social events, such as meeting friends, or eating out	1316 (58.3)	317 (59.8)	260 (50.3)	370 (59.1)	369 (63.0)	19.56	<0.001
Increased the amount I go into shops	1105 (48.9)	284 (53.6)	197 (38.1)	316 (50.5)	308 (52.6)	32.53	<0.001
Sent one or more of my children to school or pre-school	971 (43.0)	256 (48.3)	241 (46.6)	261 (41.7)	213 (36.3)	19.85	<0.001

*Values represent frequencies and percentages [n (%)] of people of answered yes, χ^2^, chi-square; P-values based on a = 0.05 level of significance following chi-square test.*

### Dietary and Lifestyle Changes After Easing the Restriction

Table 4 presents a description of dietary and lifestyle behaviors after easing the restriction among the study population. Most of the participants consumed mainly homemade meals (88.6%). For most of the dietary and lifestyle behaviors, the majority of participants reported no change: food intake (56.9%), vitamin-rich food intake (59.6%), supplement intake (60.3%), number of meals per day (66.3%), consumption of fruits and vegetables (57.9%), consumption of fast food (45.5%), consumption of fried foods (55.6%), having meals with family and friends (55.0%), physical activity level (43.8%), sleep quality (45.3%), and energy level (40%). However, most participants reported an increase in the time spent behind the screen for work (50.1%) and fun (42.9%).

**TABLE 4:** Dietary and lifestyle behaviors after easing the restriction (*n* = 2259).

Characteristics	*n* (%)
**Most consumed meals during the week**	
Homemade	2,001 (88.6)
Frozen ready-to-eat meals	27 (1.2)
Fast food	132 (5.8)
Restaurants	57 (2.5)
Healthy restaurants	42 (1.9)
**Food intake**	
Increased	676 (29.9)
Decreased	298 (13.2)
Unchanged	1,285 (56.9)
**Vitamin-rich foods intake**	
Increased	752 (33.3)
Decreased	160 (7.1)
Unchanged	1,347 (59.6)
**Supplements intake**	
Increased	732 (32.4)
Decreased	165 (7.3)
Unchanged	1,362 (60.3)
**Number of meals per day**	
Increased	484 (21.4)
Decreased	277 (12.3)
Unchanged	1,498 (66.3)
**Consumption of fruits and vegetables**	
Increased*	785 (34.7)
Decreased	165 (7.3)
Unchanged	1,309 (57.9)
**Consumption of fast foods**	
Increased	373 (16.5)
Decreased*	858 (38.0)
Unchanged	1,028 (45.5)
**Consumption of fried foods**	
Increased	325 (14.4)
Decreased*	677 (30.0)
Unchanged	1,257 (55.6)
**Meals with family and friends**	
Increased*	546 (24.2)
Decreased	470 (20.8)
Unchanged	1,243 (55.0)
**Consume breakfast daily**	
Yes*	1,331 (58.9)
No	928 (41.1)
**Skip meals**	
Yes	1,169 (51.7)
No*	1,090 (48.3)
**Snack between meals**	
Yes	1,360 (60.2)
No	899 (39.8)
**Water consumption**	
Less than eight cups (<2 l)	1,346 (59.6)
Eight cups or more (≥2 l)*	913 (40.4)
**Physical activity level**	
Increased*	580 (25.7)
Decreased	690 (30.5)
Unchanged	989 (43.8)
**Screen time for work**	
Increased	1,132 (50.1)
Decreased	311 (13.8)
Unchanged	816 (36.1)
**Screen time for entertainment**	
Increased	970 (42.9)
Decreased*	479 (21.2)
Unchanged	810 (35.9)
**Sleep quality**	
Improved*	422 (18.7)
Worsened	813 (36.0)
Unchanged	1,024 (45.3)
**Energy level**	
Improved*	462 (20.5)
Worsened	893 (39.5)
Unchanged	904 (40.0)

*Values represent frequencies and percentages [n (%)].*

*Considered as positive changes which were added to calculate the score.

Table 5 shows the association between sociodemographic confounding factors and positive dietary and lifestyle changes after easing of restrictions. The multivariate regression analyses revealed that older participants (β = 0.015, CI: 0.006–3.381; *p* = 0.001), those with higher education (β = 0.531, CI: 0.185–0.876; *p* = 0.010), and residents of Lebanon (β = 0.223, CI: −0.035–0.481; *p* < 0.001), were more likely to have a higher positive score. On the other hand, the participants who were not working from home or were unemployed (β = −0.170, CI: −0.370–0.030, and β = −0.417, CI: −0.752 to −0.082, respectively) were more likely to have a lower positive score compared to those working from home (*p* = 0.040).

**TABLE 5:** Association between positive dietary and lifestyle change score and sociodemographic characteristics in the study population (*n* = 2259).

Parameter	Positive dietary and lifestyle change score					
	Crude β	95% CI	*p*-value	Adjusted β	95% CI	*p*-value
**Age (years)**	0.012	0.005–0.019	0.001	0.015	0.006–3.381	0.001
**Sex (reference: male)**			0.239			
Female	−0.126	−0.336–0.084				
**Marital status (reference: single)**			0.632			
Married	−0.026	−0.207–0.156				
Divorced/Widowed	0.252	−0.318–0.823				
**Education level (reference: up to high school)**			<0.001			0.010
College/Bachelor’s degree	0.293	0.019–0.566		0.339	0.063–0.614	
Higher than bachelor’s degree	0.653	0.320–0.987		0.531	0.185–0.876	
**Employment status (reference: unemployed)**			0.012			0.787
Employed	0.277	0.055–0.50		0.081	−0.0151–0.314	
Student	0.012	−0.231–0.255		0.067	−0.223–0.357	
**Working/studying from home (reference: yes)**			0.028			0.040
No	−0.161	−0.0358–0.036		−0.170	−0.370–0.030	
Not applicable	−0.437	−0.768 to −0.107		−0.417	−0.752 to −0.082	
**Have chronic disease (reference: no)**			0.121			
Yes	0.227	−0.060–0.515				
**Previous COVID-19 infection (reference: no)**			0.069			
Yes	−0.172	−0.358–0.013				
**Received vaccine (reference: no)**			0.018			0.215
Yes	0.304	0.051–0.557		0.167	−0.097–0.431	
**Country of residence (reference: United Arab Emirates)**			<0.001			<0.001
Jordan	−0.375	−0.635 to −0.114		−0.403	−0.665 to −0.140	
Palestine territories	−0.471	−0.720 to −0.222		−0.276	−0.537 to −0.014	
Lebanon	0.088	−0.165–0.340		0.223	−0.035–0.481	

*CI, confidence interval; P-values based on a = 0.05 level of significance following generalized linear models analyses.*

## Discussion

The results of the study revealed that over one-third of the study participants reported weight gain since the start of the pandemic and a similar percentage have increased their food intake. A recent systematic review on the effect of the pandemic on body weight concluded that confinements during the pandemic were associated with both weight gain and weight loss (38). The review found that predictors of weight gain during the pandemic were pre-existing overweight status, emotional eating, poor sleep, and decreased physical activity (38). Moreover, data from the MENA region showed that about 40% of the adults were not engaged in physical activity and 63% had sleep disturbances during the pandemic (7). In the current study three-quarters of the participants reported unchanged or decreased physical activity levels and over one-third stated that their sleep quality got worse. This suggests that even after easing restrictions physical activity and sleep quality remained poor and in need of urgent interventions. Physical activity was also shown to decrease the mental health burden related to the COVID-19 pandemic (39). This suggests that weight gain, poor dietary choices, and physical inactivity are not specifically linked to quarantine but rather a subsequent effect of the COVID-19 pandemic. This indicates a strong need for policy action to facilitate making healthier dietary and physical activity choices.

### Attitude Toward the COVID-19 Vaccine

Surprisingly, over 85% of the participants received at least one dose of the vaccine and 78% were fully vaccinated. These rates are higher than the percentage of fully vaccinated populations reported in the same countries according to the WHO reports: United Arab Emirates 91%, Jordan 38%, Palestinian territories 29%, and Lebanon 28% (12). These values also contradict studies from the region on willingness to accept the COVID-19 vaccine (18–20). In Lebanon and Jordan, only a quarter of the participants were willing to take the COVID-19 vaccine when it becomes available (18, 19), whereas in the United Arab Emirates 60% were willing to take the vaccine (20). However, many of these studies were conducted earlier in the pandemic and before the availability of the vaccine. Although participants in the current study were concerned about the side effects of the COVID-19 vaccine and inadequate testing, they had positive attitudes toward it. The highest prevalence of vaccine hesitancy in this study was found in Palestine territories. A study that evaluated factors behind the unwillingness to receive vaccinations in Palestine territories suggested two main reasons for this, lack of vaccine evaluation and the possible long-term side effects (40). Moreover, many individuals obtain vaccine-related information from social media platforms (41). Younger age and lower education were also predictors of vaccine hesitancy (40, 42).

### Behavioral Responses After Easing the Restriction

It was hypothesized that vaccine availability and relaxation of restrictions may reduce safety measures among participants. More than half of the sample in this study reduced the frequency of disinfecting objects and joined social events. Although the direct mode of transmission of the COVID-19 virus is *via* person-to-person contact, the transmission may also occur indirectly from the objects used by the infected person (43). It is believed that the relative risk of fomite transmission is lower than direct contact or airborne transmission as many factors affect the efficiency of environmental transmission (44). Therefore, it is not clear what percentage of COVID-19 infections are obtained through fomite-mediated transmission. With the identification of new VOCs, the WHO continues to encourage authorities to strengthen public health and social measures as they have shown efficacy in reducing COVID-19 cases, hospitalizations, and deaths (1). These measures include, but are not limited to, frequent hand hygiene, use of masks, avoiding mass gatherings, physical distancing, limiting travel, and avoiding the use of public transportation (45). Moreover, it is predicted that in the absence of such measures, the vaccination program would be too slow to reduce infection and might not reduce the burden of COVID-19 effectively (46). Further studies should investigate the implications of these findings to understand how the relaxation of restrictions may be contributing to the development of new behaviors and habits.

### Dietary and Lifestyle Changes After Easing the Restriction

The findings of this study showed that many dietary and lifestyle behaviors were unchanged after easing the restriction. The MENA region is generally experiencing a rise in diet-related disorders (47) which should be attenuated regardless of COVID-19 pandemic or epidemic status. In addition, greater COVID-19 severity was observed among obese patients and patients with chronic diseases (48). On the other hand, an increase in screen time for work and entertainment was reported by most participants. A growing body of literature concerns the increased use of screens and its associated negative health outcomes. A study on families in Canada reported a 74% increase in screen time among mothers, 61% among fathers, and 87% among children (49). Similarly, studies from the MENA region, United Arab Emirates, Lebanon, Palestine, and Jordan have revealed longer screen time during the COVID-19 pandemic (9, 28–31). Excessive screen use is especially harmful to children and adolescents as it was found strongly associated with greater adiposity, unhealthy dietary habits, depressive symptoms, and reduced quality of life (50). Moreover, a recent study reported a significant association between increased screen time and higher consumption of alcohol and sweetened foods among adults (51) which are eventually energy-dense foods.

In the current study, predictors of positive dietary and lifestyle changes after easing of restrictions were older age, higher education, and working from home. Similarly, a study conducted in the United Arab Emirates showed that older adults were less likely to adopt unhealthy dietary and lifestyle habits during the pandemic (29). Moreover, a study conducted in Spain revealed that older participants and those with higher education levels had higher adherence to healthy dietary habits during the pandemic (52). Furthermore, higher educational level was associated with higher socioeconomic status which was in turn related to a better diet quality (53). The majority of participants in this study reported mainly consuming home-cooked meals. Thus, it is speculated that working from home provided them with more time to prepare home-cooked meals. Similarly, Mexican adults perceived that their dietary habits improved during the pandemic due to working from home and eating homemade food (54). Moreover, a cohort study concluded that eating home-cooked meals was associated with older age, higher socioeconomic status, and not working overtime (55). Given that positive dietary and lifestyle changes were associated with working from home, remote working should be made an option if possible to support healthful pandemic recovery.

### Strengths and Limitations

This study has several limitations. Its cross-sectional design does not allow to infer causality.

The use of a self-reported questionnaire could introduce respondent bias or data misreporting. Another potential limitation of the study might be due to the convenience sampling method used to recruit the participants, as it may produce selection bias. Moreover, a higher percentage of females completed the survey which might have impacted the generalizability of the result. Nevertheless, the use of an online survey allowed data collection from different Arab countries and covered a good sample size from each country. It also guaranteed the anonymity of the participants, thus reducing the chance of social desirability bias. The present study offers unique insights about behavioral changes after the availability of the vaccine in selected Arab countries.

## Conclusion

Overall, our findings revealed a high percentage of vaccination among the participants despite concerns about the safety and inadequate testing of the vaccines against COVID-19. Moreover, most participants joined social gatherings and reduced the frequency of disinfecting after the availability of the COVID-19 vaccines. Most participants reported no change in their dietary and lifestyle behaviors after easing the restrictions. Moreover, the results of the study revealed that older age, higher education, and working from home were associated with positive dietary and lifestyle changes.

Further explorations are needed to examine the subsequent and long-term effects of the pandemic on dietary habits, physical activity, and lifestyle changes, especially after easing restrictions. Moreover, implementing strategies to support healthful lifestyle and eating habits (e.g., working from home, social marketing) is essential to ensure that the negative impact of the pandemic does not remain in the future.

## Data Availability Statement

The datasets presented in this study can be found in online repositories. The names of the repository/repositories and accession number(s) can be found below: Figshare: https://doi.org/10.6084/m9.figshare.17890193.

## Ethics Statement

The studies involving human participants were reviewed and approved by the University of Sharjah Research Ethics Committee (Ref. REC-21-10-27-1) and Institutional Review Board of the Jordan University of Science and Technology (Ref. 33/142/2021). The patients/participants provided their written informed consent to participate in this study.

## Author Contributions

LC, TO, and ASA conceptualized and designed the project. LC, TO, ASA, MM, and SS prepared the original protocol. LC, MM, and SS did data management and analysis. LC, TO, MM, AA, DA, HIA, HA, HH, HH, LS, MH, MA, RQ, RS, RA, SS, and ASA collaborated in the overall implementation and data collection of the project. LC, MM, ASA, and SS wrote the original report with input from all co-authors. LC, TO, MM, AA, CH-M, DA, HIA, HA, HH, HH, LS, MH, MA, RQ, RS, RA, SS, and ASA read the report and made suggestions on its content. All authors approved the final manuscript.

## Conflict of Interest

The authors declare that the research was conducted in the absence of any commercial or financial relationships that could be construed as a potential conflict of interest.

## Publisher’s Note

All claims expressed in this article are solely those of the authors and do not necessarily represent those of their affiliated organizations, or those of the publisher, the editors and the reviewers. Any product that may be evaluated in this article, or claim that may be made by its manufacturer, is not guaranteed or endorsed by the publisher.
