# Supplementary material for: Assessment of Dietary and Lifestyle Responses After COVID-19 Vaccine Availability in Selected Arab Countries
Source: Front Nutr. 2022 Apr 14;9:849314. doi: 10.3389/fnut.2022.849314 (PMC9048021; doi:10.3389/fnut.2022.849314)
Supplement: Supplementary file 1 [file Data_Sheet_1.docx]

Supplementary Material

Study Questionnaire

**Title: Assessment of Dietary and Lifestyle Responses after COVID-19 Vaccine Availability in Selected Arab Countries**

**Section 1: Personal data**

**What is your gender?**

- Female
- Male

**How old are you in years?**

- ____________

**Which country do you reside in? (*drop-down “UAE, Lebanon, Jordan, Palestine”)***

- ____________

**What is your marital status?**

- Married
- Single
- Divorced
- Widowed

**What is your education Level?**

- Less than high school
- High School
- College/ Diploma
- University Degree
- Higher education masters/doctorate

**What is your employment status?**

- Full-time employment
- Part-time employment
- Unemployed
- Retired
- Self-employed
- Student

**Do you currently work/study from home?**

- Yes
- No
- Not applicable

**Did your weight change during the coronavirus pandemic?**

- Lost weight
- Gained weight
- Maintained weight

**Do you suffer from a chronic condition/disease (ex. Diabetes, Hypertension,)?**

- Yes
- No

**Have you been infected by the COVID-19 disease?**

- Yes
- No

**Have you received the COVID-19 vaccine?**

- Yes, I have completed a 2-dose vaccination **(*move to vaccine questions)***
- Yes, I have completed a one-dose vaccination **(*move to vaccine questions)***
- No, but I am planning to take it **(*move to section 2)***
- No, I do not want to take it **(*move to section 2)***

**COVID-19 vaccine**

**Did you experience any adverse reaction after receiving the vaccine?**

- Yes
- No

**If yes, what did you experience?** (*Select all that apply*)

- Redness
- Soreness
- Swelling
- Chills
- Fatigue
- Joint pain
- Headache
- Mild fever
- Other, specify _____________

**Why did you take the vaccine?**

- It was mandated by my work
- To protect against COVID-19 infection
- To travel
- To avoid restriction
- To do less frequent testing
- To stop using masks

**Section 2: Attitude toward the COVID-19 vaccine**

1. **I think the current COVID-19 situation is serious.**

- Agree
- Neutral
- Disagree

1. **I do not understand what is happening with the COVID-19 pandemic.**

- Agree
- Neutral
- Disagree

1. **I think that whether I get the coronavirus or not is out of my control**

- Agree
- Neutral
- Disagree

1. **In my opinion, people are still going to be catching the coronavirus**

- Agree
- Neutral
- Disagree

1. **Only people who are have underlying medical problems should be vaccinated**

- Agree
- Neutral
- Disagree

1. **The COVID-19 vaccine will protect me from coronavirus infection.**

- Agree
- Neutral
- Disagree

1. **I am concerned about the side effects of the COVID-19 vaccine.**

- Agree
- Neutral
- Disagree

1. **I am concerned that the vaccine has not been tested adequately.**

- Agree
- Neutral
- Disagree

1. **The COVID-19 vaccine will stop the spread of coronavirus.**

- Agree
- Neutral
- Disagree

**Section 3: Behavioral responses to the COVID-19 pandemic**

| **After easing the restriction, I have ...** | **Yes** | **No** | **Unchanged** |
| --- | --- | --- | --- |
| 1. **Washed my hands with soap and water less often than usual** |  |  |  |
| 1. **Used alcoholic hand gel less than usual** |  |  |  |
| 1. **Reduced the amount I clean or disinfect objects that I might touch, such as doorknobs, grocery, etc.** |  |  |  |
| 1. **Been in crowded places generally** |  |  |  |
| 1. **Increased the amount I use public transport** |  |  |  |
| 1. **Joined more social event, such as meeting friends, eating out, or going to a sports event** |  |  |  |
| 1. **Increased the amount I go into shops** |  |  |  |
| 1. **Sent one or more of my children to school or pre-school** |  |  |  |

**Section 4: Dietary and lifestyle changes after the availability of the COVID-19 vaccine**

**Please answer the following questions about your daily habits after easing the restriction**

1. **Most of your consumed meals during the week are?**

- Homemade
- Frozen ready-to-eat meals
- Fast food
- Restaurants
- Healthy food Restaurants

1. **Your food intake was**

- Increased
- Decreased
- Unchanged

1. **Your intake of certain foods rich in zinc and vitamins C, A, and D**

- Increased
- Decreased
- Unchanged

1. **Your intake of supplements (zinc, selenium, vitamin C, vitamin A, vitamin D)**

- Increased
- Decreased
- Unchanged

1. **Number of** **meals you eat per day**

- Increased
- Decreased
- Unchanged

1. **Your consumption of fruits and vegetables**

- Increased
- Decreased
- Unchanged

1. **Your consumption of fast foods**

- Increased
- Decreased
- Unchanged

1. **Your consumption of fried foods**

- Increased
- Decreased
- Unchanged

1. **The number of meals you consume with family or friends**

- Increased
- Decreased
- Unchanged

1. **After easing the restriction, do you consume breakfast daily?**

- Yes
- No

1. **After easing the restriction, do you usually skip meals?**

- Yes
- No

1. **After easing the restriction, do you usually snack between meals?**

- Yes
- No

1. **After easing the restriction, how much water do you drink daily?**

- Less than 8 cups (< 2 liters)
- 8 cups or more (≥ 2 liters)

1. **Your physical activity level?**

- Increased
- Decreased
- Unchanged

1. **Your screen time for work**

- Increased
- Decreased
- Unchanged

1. **Your screen time for leisure /entertainment**

- Increased
- Decreased
- Unchanged

1. **Your sleep quality**

- Improved
- Worsened
- Unchanged

1. **Your energy levels**

- Improved
- Worsened
- Unchanged
